# Characterization of the MDSC Proteome Associated with Metastatic Murine Mammary Tumors Using Label-Free Mass Spectrometry and Shotgun Proteomics

**DOI:** 10.1371/journal.pone.0022446

**Published:** 2011-08-10

**Authors:** Angela M. Boutté, W. Hayes McDonald, Yu Shyr, Li Yang, P. Charles Lin

**Affiliations:** 1 Department of Cancer Biology, Vanderbilt University Medical Center, Nashville, Tennessee, United States of America; 2 Department of Biochemistry Vanderbilt University Medical Center, Nashville, Tennessee, United States of America; 3 Department of Biostatistics, Vanderbilt University Medical Center, Nashville, Tennessee, United States of America; 4 Center for Cancer Research, National Institutes of Health, Bethesda, Maryland, United States of America; University of Georgia, United States of America

## Abstract

Expansion of Gr-1+/CD11b+ myeloid derived suppressor cells (MDSCs) is governed by the presence of increasingly metastatic, malignant primary tumors. Metastasis, not the primary tumor, is often the cause of mortality. This study sought to fully characterize the MDSC proteome in response to metastatic and non-metastatic mammary tumors using label-free mass spectrometry shotgun proteomics in a mouse model with tumor cell lines, 67NR and 4T1, derived from the same tumor. 67NR cells form only primary mammary tumors, whereas 4T1 cells readily metastasize to the lungs, lymph nodes, and blood. Overall analysis identified a total of 2825 protein groups with a 0.78% false discovery rate. Of the 2814 true identifications, 43 proteins were exclusive to the 67NR group, 153 were exclusive to the 4T1 group, and 2618 were shared. Among the shared cohort, 26 proteins were increased and 31 were decreased in the metastatic 4T1 cohort compared to non-metastatic 67NR controls after filtering. MDSCs selectively express proteins involved in the γ-glutamyl transferase, glutathione synthase pathways, CREB transcription factor signaling, and other pathways involved in platelet aggregation, as well as lipid and amino acid metabolism, in response to highly metastatic 4T1 tumors. Cell cycle regulation dominated protein pathways and ontological groups of the 67NR non-metastatic group. Not only does this study provide a starting point to identify potential biomarkers of metastasis expressed by MDSCs; it identifies critical pathways that are unique to non-metastatic and metastatic conditions. Therapeutic interventions aimed at these pathways in MDSC may offer a new route to control malignancy and metastasis.

## Introduction

Breast cancer is estimated to afflict more than 200,000 women in 2010 in the Unites States (SEER Cancer Statistics, National Cancer Institute). Breast cancer metastasizes to the lymph nodes, bone, lung, liver, and finally the brain. Some therapies are highly effective in removing and preventing recurrence of primary tumors; however, metastasis, often untreatable, is the primary cause of mortality. Myeloid derived suppressor cells (MDSCs) are a subset of heterogeneous, bone marrow derived, hematopoietic cells that home specifically to the tumors and contribute indirectly to angiogenesis, growth and metastasis. These tumor infiltrating cells foster proliferation, survival, and metastasis [1]. These cells expand in the hematopoietic organs, specifically the spleen, in response to tumor burden and malignancy [2], [3], [4]. Murine MDSCs are characterized by expression of Gr1 and CD11b cell surface markers. These cells are found in the peripheral blood of cancer patients and are positively correlated to malignancy [5], [6], which suggests MDSCS have a role in tumor invasion and metastasis. MDSCs infiltrate into tumors and accumulate at the invasive front where they promote tumor angiogenesis through regulation of VEGF bioavailability as well as tumor cell invasion and metastasis via regulation of protease activity [7]. MDSCs also confer resistance to cancer therapies [8], [9]. Hence, MDSCs are a viable target for therapeutic intervention.

Although the importance of MDSCs in tumor progression and metastasis is quite evident, the molecular mechanisms by which MDSCs achieve this feat are still unclear. Mass spectrometry based proteomics is an increasingly valuable tool in discovery of novel mediators, or “biomarkers”, of disease. MDSC gene expression varies in different tumor microenvironments [10]. However, the spleen-derived population may demonstrate signatures in response to primary tumors with high degree of malignancy and metastatic potential. Label-free proteomics, spectral counting, and protein network analysis are increasingly valuable tools for identifying protein or pathways that are specific to a pathological state [11], [12]. Protein quantitation measured by peptide spectral counts and protein network assembly are widely accepted approaches in biomarker discovery and disease characterization [13], [14]. We used a simple, but robust, approach to determine potential biomarkers of MDSC proteins relevant to tumor metastasis. This model includes two distinct tumor cell lines, 67NR and 4T1, which are derived from a single mammary tumor. While these cell lines form primary tumors with equivalent growth kinetics, they differ dramatically in their metastatic potential. 4T1 cells are highly metastatic while 67NR cells are not [15], [16].

The role of MDSCs induced by tumors with varying metastatic potential might constitute a critical mechanism by which MDSCs help tumors become even more malignant. MDSCs are largely understood from an immuno-modulatory perspective since they are immunosuppressive [17]. However, they may also facilitate tumor progression and metastasis by other means. All malignant and metastatic tumors elicit an immune response and have infiltrating immune cells; thus, rigorous analysis of the MDSC proteome in response to tumors with differential metastatic potential can provide targets of great clinical utility. Determination of an array of potential MDSC biomarkers is a unique challenge. The present study has sought to initiate defining the MDSCs proteome in response to tumors with varying degrees of metastatic potential.

## Results

### Characterization of the MDSC proteome

**Figure 1:**
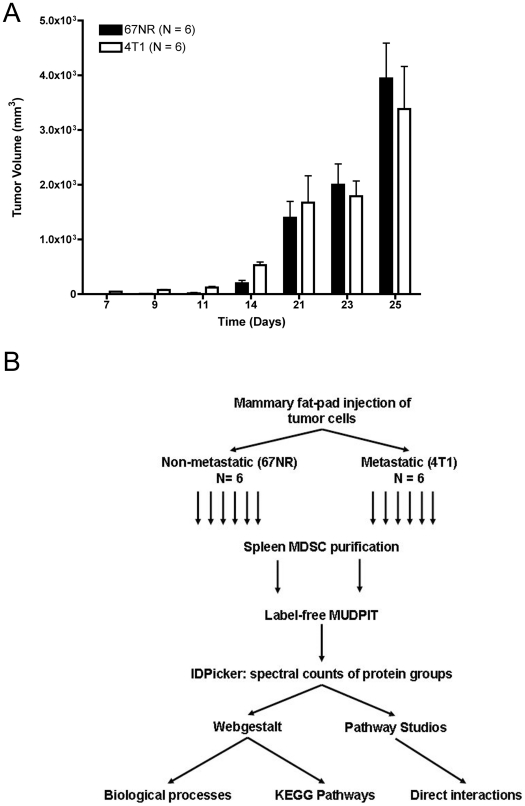
Growth kinetics of mammary tumors and proteomics experimental design. (A) Growth kinetics of mammary tumors. 500, 000 67NR or 4T1 cells were injected into the mammary fat-pad of female Balb/c AnHsd mice. The tumor volume between groups is non-significant by 2-way ANOVA. (B) Workflow outlining the semi-quantitative proteomic analysis of purified spleen MDSCs from matched non-metastatic and metastatic tumor bearing mice.

To define the MDSC proteome, we used shotgun proteomics to detect proteins that were differentially expressed in MDSCs isolated from hosts bearing non-metastatic or metastatic primary mammary tumors. 67NR and 4T1 cells, derived from the same parental mammary adenocarcinoma, differ greatly in metastatic potential although the primary tumor growth kinetics are very similar. 4T1, not 67NR, cells metastasize to lung and lymph nodes. After mammary fat-pad implantation of female Balb/c mice aged 6–7 weeks, tumor volume was monitored for 25 days. As expected, the average tumor volume was very similar by 2-way ANOVA over time and the maximum average volume reached 3942+/−645.8 (SEM) mm^3^ in the 67NR cohort and 3383+/−778.2 (SEM) mm^3^ in the 4T1 cohort (Figure 1A).

Spleen MDSCs were isolated and pooled from 6–7 mice per group then purified by magnetic cell sorting using antibodies against Gr-1 and CD11b. The two normalized MDSC populations were prepared for analysis by mass spectrometry and spectral counting as outlined in the following schema. Cells were lysed in urea/chaps buffer; proteins were precipitated with TCA, then cleaved with trypsin. Peptides were separated by strong cation exchange (SCX) directly coupled to reversed phase liquid chromatography (RP-LC) prior to direct nano-spray mass spectrometry. The resulting peptides were searched against the Uniprot database using Myrimatch and the peptides were assembled into protein groups with IDPicker. Protein groups were minimized to contain one protein each and the resulting list was then analyzed with Webgestalt and Pathway Studio (Figure 1B).

**Figure 2:**
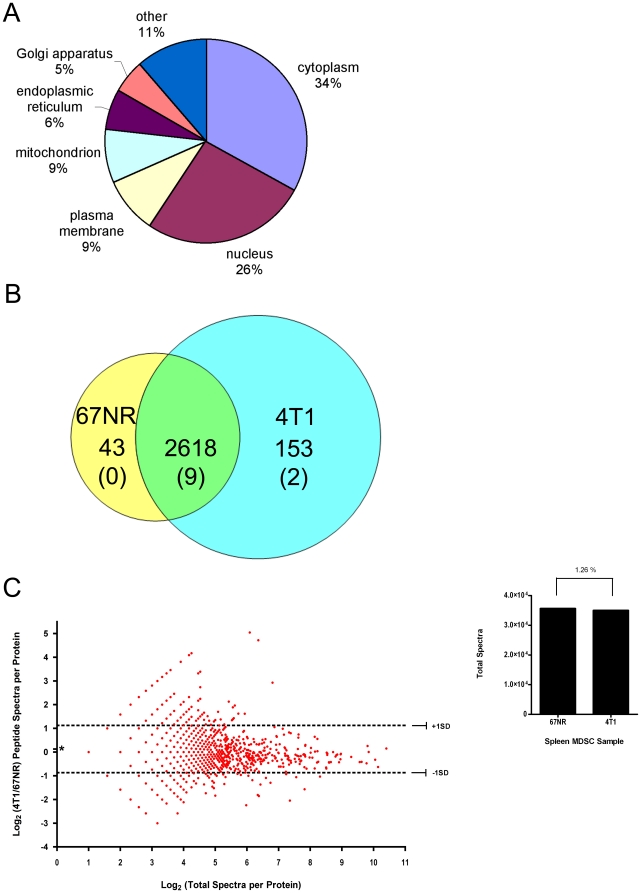
Sub-cellular distribution and quantification of the MDSC proteome in response to tumor metastatic potential. Duplicate SCX-LC-MS/MS injections of MDSCs isolated from 4T1 or 67NR tumor bearing mice were performed. (A) Each of the 2825 protein groups identified and parsed by IDPicker were analyzed using Pathway Studio and sorted by ontological group. The pie chart indicates the percentage of proteins that fall into each ontological group. (B) Union diagram of protein groups. Proteins that were shared among both groups or found exclusively in either group are indicated. The numbers of proteins found exclusively in the 67NR group (left), in both groups (middle), and exclusively in the 4T1 group (right) are indicated. The number of proteins identified from the decoy database are indicated ( ) in each MDSC group. (C) Scatter plot distribution of co-expressed MDSC proteins. The fold change is calculated as the log_2_ of spectral counts identified in MDSCs from mice with metastatic 4T1 tumors compared to those bearing non-metastatic 67NR tumors (Y-axis) compared to the total number of spectra per protein (X-axis). The average fold change 0.128 is indicated by an asterisk (*). Log_2_ fold change spectral counts that are one standard deviation from the mean are indicated by dashed lines (-·-·-) and are considered significant. Confirmation of equal loading during LC-MS/MS is indicated. The total spectral count per group, 67NR or 4T1, is indicated (inset). The coefficient of variance between the 67NR and 4T1 MDSC groups is 1.25%.

Henceforth, spleen MDSCs isolated from mice with 67NR non-metastatic or 4T1 metastatic primary tumors are identified as either 67NR- or 4T1-MDSCs, respectfully. We identified a total of 2825 proteins inclusive of 11 reverse database entries from the decoy reverse database. The false discovery rate was only 0.78%, indicating that this dataset had 99.6% (95% CI: 99.3%. 99.8%) confidence in protein identifications (Table S1). Ontological analysis of all proteins indicated that, 34%, the majority, were cytoplasmic, and 26% were nuclear. Nine percent of the protein population was derived from the plasma membrane and mitochondria each; whereas only 6% were derived from the endoplasmic reticulum and 5% from the Golgi apparatus. Eleven percent of the proteins sorted into other ontological groups (Figure 2A). After manual removal of reverse database entries, we identified 2814 proteins: 43 were unique to the 67NR-MDSCs, 153 were unique to the 4T1-MDSCs, and 2618 were detected in both groups. The number of proteins identified from the decoy database within each MDSC group is indicated by parentheses ( ) (Figure 2B).

Among the proteins shared in both 67NR and 4T1-MDSCs, the total number of spectra per protein ranges from 2, the least abundant, to 1354, the most abundant (Table S2). The fold change compared to abundance based on spectral counts is shown. Log_2_ transformation of spectral counts in 4T1-MDSCs compared to 67NR-MDSCs is indicated (Y-axis) and compared to the log_2_ transformation of total spectra per protein (X-axis, Figure 2C). The average fold change of 0.128+/−0.997 (SD) is indicated as an asterisk (*) and dashed lines (-·-·-). The range in fold change varied from −3.00 to 5.04. Therefore, all fold-change values less than −0.869 and greater than 1.125 were considered significant. Of these shared proteins, 731 or ∼26% of the proteome, exhibited a large change in abundance in the 4T1 metastatic compared to 67NR non-metastatic controls. 364 proteins were increased and 367 were decreased in 4T1-MDSCs compared to 67NR-MDSCs. To confirm the validity that protein the fold change measurements were based the metastatic potential of the primary tumor, but not sample preparation or loading; the total number of spectra from each group, 4T1 or 67NR MDSCs, was compared. 67NR-MDSCs had a total of 35,637 spectra and 4T1-MDSCs had 35,009 spectra, which indicate a coefficient of variance of only 1.26% between groups (Figure 2C, inset). The total spectral count per group is inclusive of proteins shared and unique to 67NR and to 4T1-MDSCs after removal of reverse entries.

### MDSCs specifically regulate different biological processes and pathways in response to metastasis

**Figure 3:**
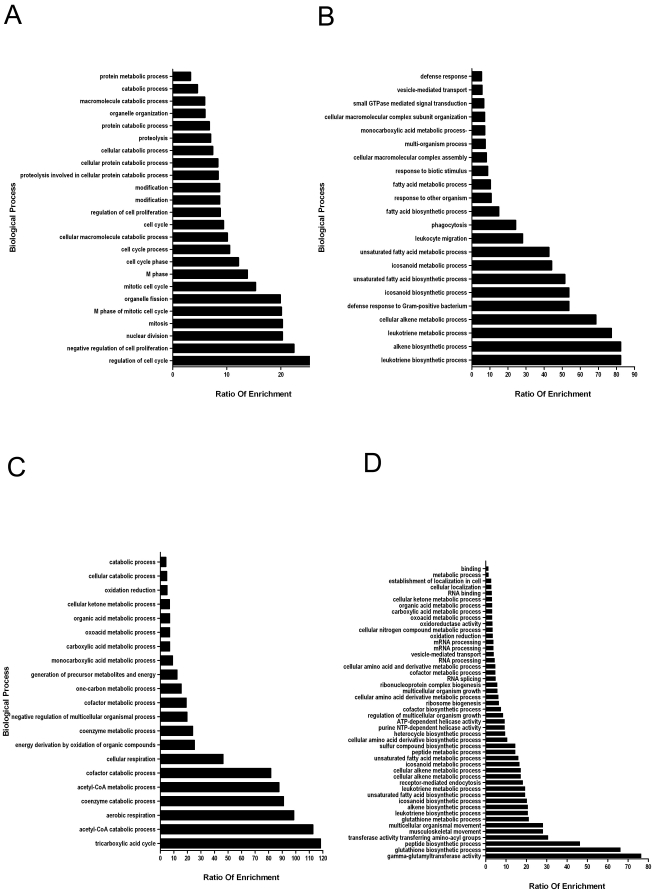
Unbiased determination of biological processes involved in MDSC response to tumor metastatic potential. Unbiased approach was used to define the most over-represented biological groups and pathways as indicated by Webgestalt. The number of proteins in each group or pathway is displayed as the “Ratio of Enrichment” (X-axis). Webgestalt generated categories (Y-axis) and the number of proteins in each pathway is displayed as the “Ratio of Enrichment” (X-axis). Categories listed on the X-axis that are most prominent values are considered the most pertinent. The protein groups used to determine enriched biological processes in each metastatic group are displayed (A) 67NR only, (B) decreased in 4T1 compared to 67NR, (C) increased in 4T1 compared to 67NR, and (D) 4T1 only.

Next, we characterized the over-represented biological processes and pathways unique to each MDSC cohort. Prior to analysis, co-expressed proteins were limited to those with 25–300 total spectra in order to exclude proteins with relatively low or excessively high abundance. Of note, proteins with very high abundance had fold change values within the standard deviation of the mean and were considered unchanged (Figure 2C). All proteins that were exclusive to either the 67NR or 4T1 MDSCs were retained for further analysis, regardless of total spectral count. Proteins were then analyzed as 4 distinct groups: (1) exclusive to 67NR-MSDCs, (2) decreased or (3) increased in 4T1 versus 67NR-MDSCs, or (4) exclusive to 4T1-MDSCs were analyzed against the mouse genome using Webgestalt. The “ratio of enrichment” (X-axis), of each protein to biological category assignment is indicated (Y-axis) is plotted for each analytical group (Figure 3). This analysis sorts and assigns each protein to categories within either biological processes or KEGG pathways and identifies the “ratio of enrichment” of each protein-category. In Webgestalt, the “ratio of enrichment” is defined as the number of proteins detected in the sample compared to the total number of proteins expected in the mouse genome in each biological process or KEGG pathway. Thus, data are internally normalized to the expected protein assignments of the reference gene set, e.g. the mouse genome.

MDSCs from 67NR and 4T1 tumor-bearing animals express proteins that represent quite different lists of biological processes (Figure 3). 67NR-MDSC proteins were primarily involved in basic cellular functions, such as M-phase cell cycle, mitosis, nuclear division, and cell cycle regulation. Interestingly, cell proliferation was negatively regulated in the 67NR group (Figure 3A). Proteins that were decreased in 4T1-MDSCs; or conversely, over-expressed in 67NR-MDSCs; were dedicated to processes involved in biosynthesis of carbon sources (alkenes), as well as fatty acids and their products, such as icosanoids and leukotrienes. Leukotriene and alkene metabolism were by far the most abundant categories (Figure 3B). In contrast, the most over-represented processes increased in 4T1-MDSCs compared to 67NR-MDSCs, were involved in cellular respiration and the TCA cycle (Figure 3C). Proteins unique to 4T1-MDSCs had, by far, the widest array of biological processes. In addition to metabolism and biosynthesis, this group is specifically characterized by helicase activity, receptor mediated endocystosis, and multicellular/musculoskeletal movement. Most surprisingly, 4T1-MSDCs selectively expressed proteins largely involved in glutathione biosynthesis and γ-glutamyl transferase activity (Figure 3D). Although there is some overlap in biological process assignments (e.g. fatty acid or icosanoid metabolism) identified from proteins decreased in or uniquely expressed in 4T1-MDSCs, the groups overwhelmingly show distinct patterns of biological processes that are unique to the presence of non-metastatic 67NR or to metastatic 4T1 tumors. In each of the four comparisons, the biological processes with the largest ratio of enrichment values are quite divergent.

**Figure 4:**
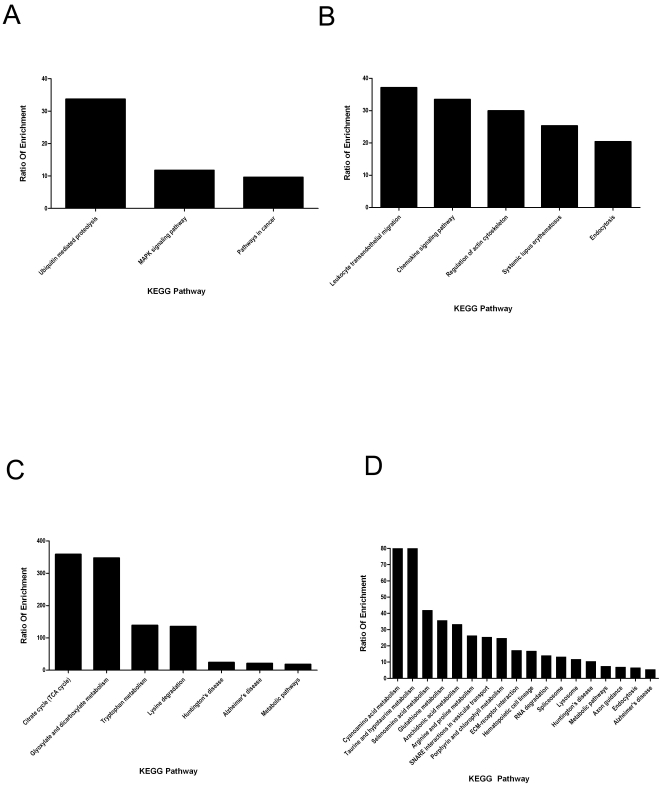
Unbiased determination of protein pathways involved in MDSC response to tumor metastatic potential. Unbiased approach was used to define the most over-represented KEGG pathways as indicated by Webgestalt. Webgestalt generated categories (X-axis) and the number of proteins in each pathway is displayed as the “Ratio of Enrichment” (Y-axis). The most prominent values are considered the most pertinent. The protein groups used to determine enriched biological processes in each metastatic group are displayed (A) 67NR only, (B) decreased in 4T1 compared to 67NR, (C) increased in 4T1 compared to 67NR, and (D) 4T1 only.

As with discovery of biological processes, assignments of MDSC proteins to KEGG pathways indicate unique events based presence of non-metastatic or metastatic tumors. MDSC proteins exclusively identified in hosts with non-metastatic 67NR tumors show regulation of ubiqutin mediated proteolysis and smaller, but unique, ratio of enrichment values involved in MAPK signaling and pathways related to cancer (Figure 4A). Proteins decreased in 4T1-MDSCs compared to 67NR-MDSCs are involved in leukocyte trans-endothelial migration, chemokine signaling, and cytoskeletal regulation (Figure 4B). These pathways are often indicators of cell survival and maintenance. The most prolific KEGG pathways detected in MDSC proteins from animals with 4T1 tumors compared to the 67NR tumors indicated enrichment of proteins largely dedicated to cellular metabolism (Figure 4C). Proteins involved in the TCA cycle and glyoxylate/dicarboxylate metabolism were by far the most represented. Metabolism of amino acids tryptophan and lysine, listed as lysine degradation, are also prominent categories. Similar to biological processes (Figure 3A–D), the proteins uniquely identified in MDSCs in the presence of 4T1 mammary tumors sorted to a much more diverse and numerous KEGG pathways (Figure 4D). Similar to proteins increased in 4T1-MDSCs compared to 67NR-MDSCs, the most prominent categories in 4T1 specific MDSCs involve amino acid metabolism. Cyano-amino acid, taurine and hypotaurine, as well as seleno-amino acids have the highest ratio of enrichment values. In addition, arginine and proline metabolism are uniquely present, albeit at lower enrichment values. Surprisingly, glutathione and arachidonic acid metabolism are specifically identified in this group, similar to biological processes (Figure 3). In addition, soluble NSF attachment protein receptor (SNARE) interactions in vesicular transport; extracellular matrix (ECM)-receptor interactions were also exclusively identified in this metastatic 4T1 cohort (Figure 4D).

### Differential MDSC protein-protein interactions in response to non-metastatic or metastatic mammary tumors

**Figure 5:**
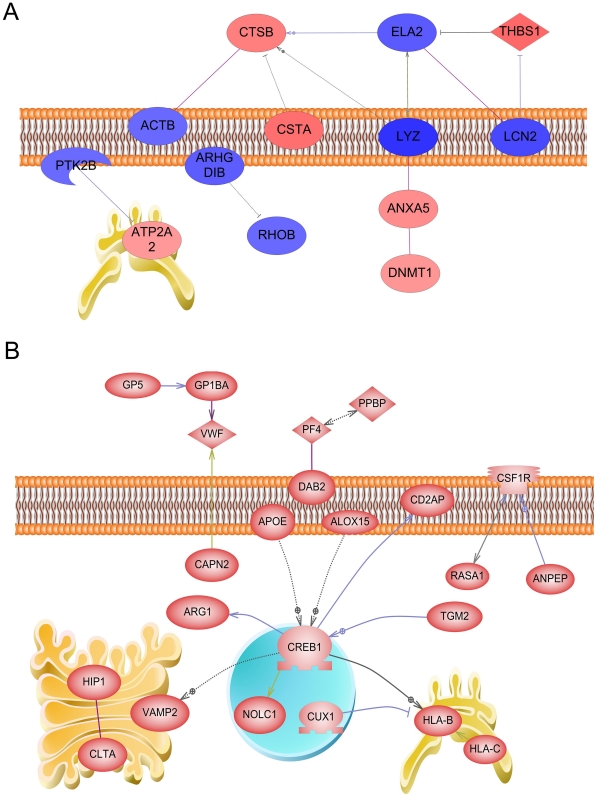
Pathway analysis of the MDSC proteome in response to non-metastatic or metastatic tumors. Candidate proteins were uploaded into the Pathway Studio program after manually filtering for protein groups with 25–300 total spectra as described. Proteins in each group were analyzed for direct interactions and localization as determined by Pathway Studio. Binding partners (solid purple line), activating proteins (grey-blue line and arrowhead), inhibitory proteins (grey line and bar) are shown. Proposed interactions are indicated by dashed lines (-·-·-) and evidence-based interactions are indicated with solid lines (—). The plasma membrane and the organelles are illustrated to indicate protein localization. (A) Differentially abundant proteins in 67NR and in 4T1-MDSCs. Proteins increased (purple to red) or decreased (blue to purple) in 4T1-MDSCs compared to 67NR-MDSCs. (B) Proteins exclusively detected in 4T1-MDSCs.

Like dominant biological processes or protein pathways, direct interactions between proteins can provide insight of valuable potential biomarkers of disease or specific pathological states. Understanding direct interactions can also provide evidence of critical biomarkers of the MDSC proteome in response to metastatic tumors. In this study, direct interactions were defined by Pathway Studio in two groups of proteins derived from spectral counting. The first analysis illustrates proteins with differential spectral counts that were increased (pink–red) and decreased (light–dark blue) in 4T1-MDSCs compared to 67NR-MDSCs (Figure 5A). The second group illustrates direct interactions between proteins specifically detected in 4T1-MDSCs (Figure 5B). The absolute spectral count and fold change value of each protein is listed (Table S2 and Table S3). All entries are based on references derived from Pathway Studio.

First, proteins that had differentially abundant spectral counts in 67NR and 4T1 groups were analyzed (Figure 5A). Neutrophil elastase (ELA2) is decreased by 1.09 fold and thrombospondin 1 (TSP1) is increased by 2.13 fold in 4T1-MDSCs. TSP1 is a negative regulator of human neutrophil elastase. This study also indicates that thrombospondin is a positive regulator of cathepsin B, detected to have a 1.62 fold increase in the 4T1-MDSCs. A known inhibitor of cathepsin B, cystatin A (identified as stefin A-like protein), is also increased by 2.53 fold. However, β-actin, a cathepsin B binding partner, is decreased by 0.93 fold in 4T1-MDSCs. Lysosyme (LYZ), a positive regulator of both cathepsin B and of ELA2, is decreased by 1.57 fold in the presence of highly metastatic 4T1 conditions. LYZ was also defined as a binding partner of annexin A5 (ANXA5) which in turn binds DNA (cytosine-5-methyltransferase 1 (DNMT1). LYZ is decreased, but the downstream proteins ANXA5 and DNMT1 are increased by 1.32 and 1.15, respectfully. DNMT1 is nuclear, but reported to co-localize with annexin V. Lipocalin 2 (LCN2) is decreased by 1.29 fold and is also negatively associated with THBS1, which is increased by 2.13 fold and identified as a binding partner of ELA2.

Ras homolog gene family, member B (RHOB) and its negative regulator, Rho GDP dissociation inhibitor (GDI) beta (ARHGDIB), and are both suppressed in 4T1-MDSCs. Our data indicated that RHOB and ARHGDIB are decreased by 0.93 and 1.06 fold, respectfully. Tyrosine kinase 2β (PTK2B) is largely decreased; a negative 0.92 fold change was detected. In contrast, ATPase, Ca++ transporting, cardiac muscle, slow twitch 2 (ATP2A2) is increased by 1.13 fold. This result was fitting since PTK2B gene expression is inversely correlated to that of ATP2A2 based on pathway analysis. The gene encoded by this ATPase, sarcoendoplasmic reticulum calcium transport ATPase (SERCA2), is inversely correlated to PTK2B expression. Thus, our finding that PTK2 is decreased and ATPase/SERCA2 is increased is in accordance with other reports defined by this protein network.

### Metastasis specific protein interactions in MDSCs

To further interpret the MDSC proteome in response to metastatic tumors, proteins uniquely identified in MDSCs from mice with 4T1 metastatic mammary tumors were analyzed using Pathway Studio (Figure 5B). Direct interactions revealed dominance of proteins primarily involved in lipid metabolism and platelet cell aggregation. In addition, neutrophil chemo-attractant proteins like colony stimulating factor receptor 1 (CSFR1) and pro-platelet basic protein (PPBP), as well as arginase I (ARGI) were specifically detected in MDSCs from mice with metastatic 4T1 tumors (Figure 5B). Glycoprotein V (GP1BA), Von Willibrand Factor (vWF), and platelet factor 4 (PF4) are also specifically found in this cohort. Herein, GP5 was positively correlated to GP1BA, then VWF. Both were upstream of disabled homolog 2 (DAB2), a mitogen-responsive phosphoprotein. In this case, DAB2 is reported to partially co-localize with Platelet Factor (PF) 4. Colony stimulating factor receptor (CSF1R) was positively correlated to aminopeptidase N (ANPEP) which, in turn, was positively correlated with RAS p21 protein activator 1 (GTPase activating protein).

Huntington interacting protein (HIP) and clatherin (CTLA) are binding partners and were specifically detected in 4T1-MDSCs. This observation was in accordance with the biological processes defined in the same sample and identified as the Huntington pathway (Figure 4). The interaction between HIP and CTLA is reported to facilitate clatherin assembly. Proteins involved in vesicular transport and/or endocytosis are specifically up-regulated in MDSCs derived from 4T1 metastatic tumor bearing mice. Interestingly, these proteins were assigned to the Golgi apparatus.

Notably, proteins previously described in these cells or those with similar lineage are also detectable in this study. Major histocompatibility complex B and C paralogues, HLA-B and HLA-C, were specifically detected and increased in 4T1-MDSCs. The nuclear protein, Cut 1 homeobox protein (CUX1), was shown to be negatively correlated to HLA. Both CUX1 and HLA-B are present in 4T1-MDSCs, but the CUX1 had 4 spectral counts and HLA-B (identified as H-2 class I histocompatibility antigen) had 13 spectral counts, respectfully (Table S2 and Table S3).

Proteins specifically identified in MSDCs from animals with 4T1 tumors, compared to those concurrently identified in mice with 67NR tumors, show very unique direct interaction pathways. Surprisingly, cAMP responsive element binding protein 1 (CAMP), had the highest degree of direct connectivity among all proteins in this study. This transcription factor was not detectable in MDSCs from the non-metastatic 67NR tumor cohort. Apolipoprotein E (APOE) and arachidonate 15-lipoxygenase (ALOX15) were identified as positive regulators of CREB. ApoE mediates the binding, internalization, and catabolism of lipoprotein particles. ALOX15 converts arachidonic and linoleic acid to metabolites, a process that occurs during leukotriene biosynthesis, which was also indicated by biological processes identified in MDSCs from this 4T1 metastatic tumor group (Figure 4D). Transglutaminase (TGM2) positively associated with CREB expression, normally functions to catalyze the cross-linking of proteins.

Many proteins were regulated by CREB in 4T1-MDSCs. Vesicle-associated membrane protein 2 (VAMP2); arginase I (ARGI), HLA-B, and CD2-associated protein (CD2AP), and nucleolar and coiled-body phosphoprotein 1 (NOCL1) are all downstream of CREB. Similar to HIP and CTLA, VAMP2 mediates vesicle docking and fusion. ARGI is well studied in MDSCs and catalyzes the hydrolysis of arginine to ornithine and urea. These data indicate a positive correlation between a ubiquitous transcription factor and vesicular Golgi transport, the urea cycle, actin cystoskeletal scaffolding, as well as antigen presentation and T-cell contact.

## Discussion

Spleenic MDSC expansion is a key characteristic and facilitator of tumor burden and metastasis. Within the immune cell population, Gr1+/CD11b+ MDSCs, are particularly vexing. The number of bone marrow and spleen MDSCs increase proportionally to metastatic potential of the primary tumor and contain two subpopulations with anti-immune effects [18], [19]. MDSC levels positively correlate with tumor malignancy [5], [6] and confer resistance to cancer therapies [20]. Understanding how MDSCs contribute to these processes is invaluable. Here, we characterized the MDSC proteome in response to a “metastatic gradient” in a mouse model of metastatic breast cancer. Duplicate MudPIT, spectral counting, and protein network analysis indicated that spleen MDSCs overwhelmingly express proteins involved in glutathione and γ-glutamyl transferase activity, in amino acid and lipid metabolism, and in angiogenic or platelet signaling in response to metastatic, but not non-metastatic, mammary tumors. These results have detected clear differences in over-represented biological processes, protein pathways, and protein-protein interactions under metastatic conditions.

### Differential expression of amino acid/lipid metabolism, nutrient depletion, and drug resistance associated pathways in malignant MDSCs

Based on the proteomic profiling, proteins in MDSC associated with malignant 4T1 tumors were primarily involved in glutathione and γ-glutamyl transferase activity, amino acid and lipid metabolism, and angiogenic or platelet signaling. Glutathione transferases, GGT1 and GGT5 are detected in 4T1-, but not in 67NR-MDSCs. GGT inhibition leads to differentiation [21]; therefore, activating the enzyme is expected to inhibit cell differentiation. MDSCs are considered immature myeloid cells – a lack of differentiation is a key feature of these cells. Since MDSCs are a heterogeneous population, it would be interesting to see whether malignant tumor cells affect MDSC differentiation differently from non-malignant tumors. In addition, GGT5 is a leukotrienase that hydrolyze GSH; and GSH was also only detectable in 4T1-MDSCs. Studies have shown that metastasis is directly proportional to GGT activity and GSH hydrolysis in B16 liver metastasis [22] and that GGT is also increased in patients with breast cancer [23]. Transglutaminase (TGM) is another protein elevated in 4T1-MDSCs identified in this study. TGM2 is associated with metastatic breast cancers and multi drug resistant tumors [24], [25]. TGM expression correlated with poor survival in colon cancer patients wherein it may be a prognostic or predictive factor [26]. Although published studies were conducted with tumor cells, we find it uncanny that the same relationship between these proteins was detected solely in MDSCs from the metastatic, not the non-metastatic, cohort. Thus, our findings reveal the existence of potential mediators in MDSCs responsible for metastasis.

ApoE, another protein specifically identified in 4T1-MDSCs, has been associated with depth of tumor invasion, degree of lymph node metastasis, and with clinical stage. It is also increased in tumors compared to normal tissues [27]. In contrast, ALOX proteins in the 4T1 cohort are reduced compared to cells from non-malignant hosts. Over-expression of ALOX suppresses the myeloid cell development [28]. Thus, reduced spectral count for ALOX may indicate increased MDSC expansion in response to metastasis.

As aforementioned, multiple studies have reported that MDSCs confer resistance to cancer therapies. Surprisingly, we found increase of GSH in 4T1 MDSCs. Cleavage of GSH generates cystenyl glycine that binds to cisplatin, a chemotherapeutic agent, making it non-permeable to the cell membrane [29]. Reduced efficacy of cisplatin occurs with GGT, another protein we discovered associated with 4T1 tumors host, over-expression and may be a mechanism chemotherapy resistance. Although many of these mechanisms have been studied in tumor cells, it is quite interesting to note that MDSCs are also a source of such proteins in mouse models with highly metastatic tumors. This result suggests that malignant tumor cells activate lipid and amino-acid metabolism and GGT/GSH pathways within MDSCs to facilitate or exacerbate tumor metastasis.

Another major difference between 4T1-MDSCs and 67NR-MDSCs is indicated by proteins involved in metabolism and energy expenditure. In particular, ATP2A2, or SERCA2, is increased while PTK2B is decreased. SERCA2 mRNA is shown over-expressed in cancerous tissues compared to normal tissue, which is also positively correlated to invasion and metastasis. In addition, high SERCA2 is associated with poor clinical outcome and metastasis in cancer patients [30]. SERCA2b, another ATPase, decreases with retinoic acid induced differentiation [31] indicating there is an inverse relationship between immune cell differentiation and ATPases. Together, the data show 4T1 MDSCs had a specific increase in proteins involved in cellular metabolism and use of multiple amino acids or lipids as carbon sources. Increased metabolism within and nutrient depletion by MDSCs has been reported [32], [33].

CREB, a transcription factor, was specifically detected in 4T1-MDSCs and had the highest degree of connectivity. CREB is highly expressed in myeloid progenitors, a cell population including MDSCs. CREB transgenic mice have uncontrolled myelopoeisis [34]. In addition, CREB is positively correlated to tumor malignancy and progression, as well as angiogenesis in a model of leukemia [35]. Based on this evidence, it is reasonable to suggest that malignant tumors upregulate CREB in MDSCs that regulate the expansion and differentiation of MDSCs in order to promote tumor metastasis.

### Pro-angiogenic, platelet-like pathways and proteases are altered in MDSCs associated with metastatic tumors

We detected proteins differently expressed in 4T1-MDSCs are involved in angiogenesis and signaling common to platelets, such as LCN2 which is a neutrophil marker, is also known as neutrophil gelatinase-associated lipocalin. Lipocalin inhibits ras-induced tumor angiogenesis through the down-regulation of VEGF expression and the up-regulation of THBS1 expression in tumor cells [36]. Our data show that lipocalin and its binding partner ELA2 are lost, while cathepsin B was increased, in MDSCs associated with 4T1 tumors. Of note, decreased ELA2 is associated with greater metastasis of primary tumors [37], [38]. This expression pattern is consistent with the metastatic property of 4T1 tumors, since lipocalin is negatively associated to angiogenesis and metastasis [39], while cathepsin B is positively correlated to tumor growth and metastasis [40], [41], [42]. Tumor derived cathepsin B is quite often associated with tumor cell invasion and increased metastasis. These findings indicate a comprehensive effect of malignant tumor upon MDSCs. Metastatic tumors up-regulate proteases and down regulate angiogenic inhibitors in MDSCs to promote tumor cell invasion and metastasis.

A group of proteins associated with angiogenesis and with platelets are specifically elevated in 4T1-MDSCs. For example, calpains, cysteine proteases, cleave vWF and increase platelet cell aggregation [43], [44]. Platelet glycoprotein V (GP5) constitutes the vWF receptor and mediates adhesion. Glycoprotein Ib (GP1BA), a vWF receptor, also initiates signaling of platelet activation, thrombosis, and hemostasis. It is known that platelet-tumor interaction through vWF facilitates extravasation through the blood vessels and results in greater metastases [45], [46]. Lastly, PPBP, a CXC chemokine domain containing protein, and PF4 were also detected in our study. Both proteins are neutrophil chemoattractants and PF4 may have roles in inflammation, angiogenesis, and platelet aggregation [47]. Perhaps all of these proteins in MDSCs assist in lymphocyte, endothelial cell, and tumor cell adhesion during metastatic tumor development, harboring processes that encompass innate immunity, extravasation, and tumor infiltration.

Cystatin A, a well-known inhibitor of cathepsin B, is also increased in 4T1 MDSCs, which is contrary to expectations. Interestingly, neutrophilic granule protein (NGP), a potential cathepsin B inhibitor, is also decreased as reported in a similar study (Table S2 and Table S3) [48]. THBS1 is another protein increased in MDSCs from the 4T1 tumor-bearing host. THBS1 is an endogenous angiogenic inhibitor that regulates endothelial cell apoptosis, protease expression and angiogenic factor expression. Elevated THBS1 expression in MDSCs of 4T1 tumor host seems contradictory to the metastasis phenotype of aggressive tumor cells. Yet, the relationship of this protein with metastasis is controversial. Findings point to both negative and positive roles of THSB1 in metastasis [49], [50], suggesting the activity of THSB1 in tumor progression is dependent on its interactions with several host and tumor associated factors, as well as cell type. This seemingly conflicting data of protein expression and biological phenotype warrant further investigation.

### MDSCs polarity and immune toxicity

A cytoskeletal protein, CD2AP, is specifically detected in MDSCs in response to 4T1 metastatic tumor burden. CREB, increased in 4T1 MDSCs, is reported to trans-activate the CD2AP promoter and CD2AP has been reported to interact with both membrane and actin cytoskeletal proteins in order to facilitate cytoskeletal polarity between antigen presenting cells and T-cells [51]. MDSC is also called an immature dendritic cell and possesses immune suppressive activity. Cross-talk and cell-cell contact may aid MDSCs' ability to subvert immune surveillance in highly metastatic cancer.

Arginase I, another elevated protein in 4T1 MDSCs, is known to deplete cystine and cysteine that T-cells require for activation and function. This depletion is a mechanism by which MDSCs kill T cells [52]. Changes in pathways involved in amino acid metabolism and arginase I activity suggest that MDSCs are, in fact, more harmful to the innate immune response when the host is burdened with highly metastatic tumors compared to non metastatic tumors. Evidence of this phenomenon is presented in many studies showing that MDSCs isolated from metastatic models compared to non-metastatic controls are advantageous to tumor development and metastasis [53], [54].

### Conclusions

MDSCs, which promote tumor growth and metastasis, are dramatically increased in cancer patients and animal models. MDSC levels are positively correlated with tumor malignancy. This study determined the proteome of MDSCs associated with malignant and metastatic tumor conditions. The MDSC proteome responded quite differently to non-metastatic and metastatic mammary tumors. In response to metastatic tumors, MDSC proteins were primarily involved in glutathione and γ-glutamyl transferase activity, lipid and amino acid metabolism, and signaling pathways involved in platelet aggregation and angiogenesis. This study further implicates that MDSCs may be part of a pro-metastatic feedback loop. Targeting MDSCs to disrupt these pathways may provide a therapeutic intervention during metastatic tumor treatment. This report provides not only a network of MDSC proteins that are influenced by the metastatic tumor, but may have identified novel biomarker candidates and pathways that warrant further study.

## Materials and Methods

### Chemicals and Cell Lines

All chemical reagents were purchased from Sigma-Aldrich (St. Louis, MO). Mouse mammary 67NR and 4T1 tumor cells were maintained in DMEM supplemented with 10% FBS and 1% penicillin-streptomycin.

### Animal Model and MDSC Purification

All mice used in this work were housed in pathogen-free units at Vanderbilt University Medical Center, in compliance with IACUC regulations. This study protocol (ID# M/05/083) was approved by the Chairperson of IACUC, the Office of Animal Welfare, at Vanderbilt University Medical Center. 6–7 week old female Balb/c mice were purchased from Harlan Laboratories (Indianapolis, IN). 500,000 67NR or 4T1 cells per mouse were injected into the 4th mammary fat-pad as described [7]. Single cell suspensions were prepared from fresh spleen tissue harvested from mice with 4T1 and 67NR primary tumors as described [14]. Gr1+/CD11b+ cells were sequentially selected with magnetic anti-Gr1 and anti-CD11b antibody-beads (Miltenyi Biotec, Auburn, CA). After 25 days of primary tumor growth, spleens were collected and pooled. Gr1+/CD11b+ MDSCs from mice with non-metastatic (67NR) or metastatic mammary tumors (4T1) were isolated from spleenocytes using magnetic associated cell sorting and positive selection with anti-Gr1 and anti-CD11b antibodies.

### Sample Preparation, Mass spectrometry and Spectral Counting

Purified cells were normalized by cell count and lysed in urea buffer (7M urea, 1M thiourea, 4% CHAPS, 4 mg/mL DTT, pH 8.0). Protein was precipitated using 25% TCA (w/v), washed with ice cold acetone, and resuspended in 8M urea 100 mM Tris, pH 8.0. Proteins were reduced with TCEP, alkylated with iodoacetemide, then digested with trypsin. The resulting peptide mixtures were split into duplicates and analyzed via MudPIT similar to the methods described [55]. Briefly, peptides were loaded onto a biphasic pre-column fritted using an Upchurch M-520 filter union. This 150 µm fused silica microcapillary column was packed with 5-µm C_18_ reverse-phase resin followed by strong cation-exchange resin. Once loaded, it was then placed in-line with a 100 µm×20 cm, C_18_ packed emitter tip column coupled to an LTQ ion trap mass spectrometer nanospray source. All columns and resins were purchased from Phenomenex (Torrance, CA). Separations were accomplished using 25–100 mM pulses of ammonium acetate followed by a 115 min reversed phase gradient of 0–40% acetonitrile 0.1% formic acid. Tandem mass spectra were collected in a data dependent manner using dynamic exclusion. Spectra were extracted using ScanSifter (Vanderbilt University, Nashville, TN) and searched against the mouse Uniprot database that also contained reversed versions of the proteins using Myrimatch and peptide to protein matches and spectral counts were assembled using IDPicker [56], [57] using a false discovery rate maximum of 5%. All reported proteins were identified with a minimum of 2 distinct peptides. Sample loading was confirmed by IDPicker and displayed as the total number of spectra per group. The fold change of each protein was calculated by comparing the log_2_ of total spectra per protein compared to the log_2_ ratio of spectra in 4T1 derived MDSCs compared to those from 67NR derived MDSCs.

### Gene Ontology and Pathway Analysis

Among proteins identified in both 67NR and 4T1 cohorts, the average spectral count change +/− one standard deviation from the average fold change of all proteins in the dataset was calculated. To simplify large datasets, all proteins that met the filter criteria by IDPicker and identified in both 67NR and 4T1 groups were filtered to include proteins containing 25–300 total spectral counts. All proteins exclusively detected in either 67NR or 4T1 groups were retained. Immunoglobulin and hemoglobin proteins were excluded from analyses. Proteins were separated into 4 analytical groups: (1) unique to 67NR, (2) decreased in 4T1 compared to 67NR, (3) increased 4T1 compared to 67NR, (4) and unique to 4T1 prior to analysis by Webgestalt (Vanderbilt University, Nashville, TN) [15] and Pathway Studio version 7.1 (Ariadne Genomics, Inc., Rockville, MD). In Webgestalt, each group of proteins were compared to the mouse genome reference set and assigned to Gene Ontology (GO) biological processes or to the Kyoto Encyclopedia of Genes and Genomes (KEGG) using the hypergeometric test for enrichment analysis with the Benjamini-Hochberg for multiple test alignment. The significance cut-off was p<0.05 and a minimum of 2 proteins were accepted per category. The resulting data is displayed at the “Ratio of Enrichment” compared to the entire mouse genome [58], [59], [60]. Direct interactions were defined by Pathway Studio in two cohorts: (1) proteins increased and decreased in 4T1-MDSCs compared to 67NR-MDSCs, and (2) proteins specifically detected in 4T1-MDSCs. In both cases, proteins that had no direct binding partners were removed. Default labeling of interactions was used.

### Statistical analysis

Tumor growth data was analyzed using Prism (Graph-Pad, La Jolla, CA, USA) and compared by 2-way ANOVA and values are expressed as mean +/− SEM. The protein identification false discovery rate is calculated as described [61], [62]. Spectral count data was subjected to log-log transformation and analyzed using fold change analysis. Fold change values 1 standard deviation from the mean were considered significant [63]. For all other tests, for tumor volume, and for ontological assignments; p<0.5 was considered significant.

## Supporting Information

Table S1 The false discovery rate of MDSC proteins identified by MUDPIT in response to tumor metastatic potential. *^a^* Biological group, *^b^* Total Number of Spectra, *^c^* Forward database spectral identifications, *^d^* Reverse Database Spectral Identifications, and *^e^* Calculated False Discovery Rate (%).
(XLS)

Table S2 Potential MDSC protein biomarkers in response to tumor metastatic potential. *^a^* Protein Name, *^b^* Gene Description, *^c^* Uniprot ID, *^d^* Spectral Count and, *^e^* Log_2_ (spectral count 4T1/67NR) protein abundance ratio.
(XLS)

Table S3 All MDSC proteins identified by MUDPIT in response to tumor metastatic potential. *^a^* Protein Name, *^b^* Gene Description, *^c^* Uniprot ID , *^d^* spectral count, *^e^* total spectral count, and *^f^* Log_2_ (spectral count 4T1/67NR) protein abundance ratio.
(XLS)

## References

[1] Coussens LM, Werb Z (2002). Inflammation and cancer.. Nature.

[2] Greten TF, Manns MP, Korangy F (2011). Myeloid derived suppressor cells in human diseases.. Int Immunopharmacol.

[3] Ribechini E, Greifenberg V, Sandwick S, Lutz MB (2010). Subsets, expansion and activation of myeloid-derived suppressor cells.. Med Microbiol Immunol.

[4] Youn JI, Nagaraj S, Collazo M, Gabrilovich DI (2008). Subsets of myeloid-derived suppressor cells in tumor-bearing mice.. J Immunol.

[5] Almand B, Clark JI, Nikitina E, van Beynen J, English NR (2001). Increased production of immature myeloid cells in cancer patients: a mechanism of immunosuppression in cancer.. J Immunol.

[6] Diaz-Montero CM, Salem ML, Nishimura MI, Garrett-Mayer E, Cole DJ (2009). Increased circulating myeloid-derived suppressor cells correlate with clinical cancer stage, metastatic tumor burden, and doxorubicin-cyclophosphamide chemotherapy.. Cancer Immunol Immunother.

[7] Yang L, DeBusk LM, Fukuda K, Fingleton B, Green-Jarvis B (2004). Expansion of myeloid immune suppressor Gr+CD11b+ cells in tumor-bearing host directly promotes tumor angiogenesis.. Cancer Cell.

[8] Shojaei F, Wu X, Malik AK, Zhong C, Baldwin ME (2007). Tumor refractoriness to anti-VEGF treatment is mediated by CD11b+Gr1+ myeloid cells.. Nat Biotechnol.

[9] Finke J, Ko J, Rini B, Rayman P, Ireland J (2011). MDSC as a mechanism of tumor escape from sunitinib mediated anti-angiogenic therapy.. Int Immunopharmacol.

[10] Joyce JA, Pollard JW (2009). Microenvironmental regulation of metastasis.. Nat Rev Cancer.

[11] Saydam O, Senol O, Schaaij-Visser TB, Pham TV, Piersma SR (2010). Comparative protein profiling reveals minichromosome maintenance (MCM) proteins as novel potential tumor markers for meningiomas.. J Proteome Res.

[12] Neilson KA, Ali NA, Muralidharan S, Mirzaei M, Mariani M (2011). Less label, more free: Approaches in label-free quantitative mass spectrometry.. Proteomics.

[13] Oti M, Snel B, Huynen MA, Brunner HG (2006). Predicting disease genes using protein-protein interactions.. J Med Genet.

[14] Albrethsen J, Knol JC, Piersma SR, Pham TV, de Wit M Subnuclear proteomics in colorectal cancer: identification of proteins enriched in the nuclear matrix fraction and regulation in adenoma to carcinoma progression.. Mol Cell Proteomics.

[15] Aslakson CJ, Miller FR (1992). Selective events in the metastatic process defined by analysis of the sequential dissemination of subpopulations of a mouse mammary tumor.. Cancer Res.

[16] Yang J, Mani SA, Donaher JL, Ramaswamy S, Itzykson RA (2004). Twist, a master regulator of morphogenesis, plays an essential role in tumor metastasis.. Cell.

[17] Gabrilovich DI, Nagaraj S (2009). Myeloid-derived suppressor cells as regulators of the immune system.. Nat Rev Immunol.

[18] Yang L, Huang J, Ren X, Gorska AE, Chytil A (2008). Abrogation of TGF beta signaling in mammary carcinomas recruits Gr-1+CD11b+ myeloid cells that promote metastasis.. Cancer Cell.

[19] Movahedi K, Guilliams M, Van den Bossche J, Van den Bergh R, Gysemans C (2008). Identification of discrete tumor-induced myeloid-derived suppressor cell subpopulations with distinct T cell-suppressive activity.. Blood.

[20] Shojaei F, Ferrara N (2008). Refractoriness to antivascular endothelial growth factor treatment: role of myeloid cells.. Cancer Res.

[21] Bauvois B, Laouar A, Rouillard D, Wietzerbin J (1995). Inhibition of gamma-glutamyl transpeptidase activity at the surface of human myeloid cells is correlated with macrophage maturation and transforming growth factor beta production.. Cell Growth Differ.

[22] Obrador E, Carretero J, Ortega A, Medina I, Rodilla V (2002). gamma-Glutamyl transpeptidase overexpression increases metastatic growth of B16 melanoma cells in the mouse liver.. Hepatology.

[23] Seth LR, Kharb S, Kharb DP (2003). Serum biochemical markers in carcinoma breast.. Indian J Med Sci.

[24] Mehta K, Fok J, Miller FR, Koul D, Sahin AA (2004). Prognostic significance of tissue transglutaminase in drug resistant and metastatic breast cancer.. Clin Cancer Res.

[25] Verma A, Mehta K (2007). Tissue transglutaminase-mediated chemoresistance in cancer cells.. Drug Resist Updat.

[26] Miyoshi N, Ishii H, Mimori K, Tanaka F, Hitora T (2010). TGM2 is a novel marker for prognosis and therapeutic target in colorectal cancer.. Ann Surg Oncol.

[27] Chen YC, Pohl G, Wang TL, Morin PJ, Risberg B (2005). Apolipoprotein E is required for cell proliferation and survival in ovarian cancer.. Cancer Res.

[28] Middleton MK, Zukas AM, Rubinstein T, Jacob M, Zhu P (2006). Identification of 12/15-lipoxygenase as a suppressor of myeloproliferative disease.. J Exp Med.

[29] Hanigan MH, Gallagher BC, Townsend DM, Gabarra V (1999). Gamma-glutamyl transpeptidase accelerates tumor growth and increases the resistance of tumors to cisplatin in vivo.. Carcinogenesis.

[30] Chung FY, Lin SR, Lu CY, Yeh CS, Chen FM (2006). Sarco/endoplasmic reticulum calcium-ATPase 2 expression as a tumor marker in colorectal cancer.. Am J Surg Pathol.

[31] Launay S, Gianni M, Kovacs T, Bredoux R, Bruel A (1999). Lineage-specific modulation of calcium pump expression during myeloid differentiation.. Blood.

[32] Ostrand-Rosenberg S (2010). Myeloid-derived suppressor cells: more mechanisms for inhibiting antitumor immunity.. Cancer Immunol Immunother.

[33] Srivastava MK, Sinha P, Clements VK, Rodriguez P, Ostrand-Rosenberg S (2010). Myeloid-derived suppressor cells inhibit T-cell activation by depleting cystine and cysteine.. Cancer Res.

[34] Cheng JC, Kinjo K, Judelson DR, Chang J, Wu WS (2008). CREB is a critical regulator of normal hematopoiesis and leukemogenesis.. Blood.

[35] Sandoval S, Pigazzi M, Sakamoto KM (2009). CREB: A Key Regulator of Normal and Neoplastic Hematopoiesis.. Adv Hematol.

[36] Venkatesha S, Hanai J, Seth P, Karumanchi SA, Sukhatme VP (2006). Lipocalin 2 antagonizes the proangiogenic action of ras in transformed cells.. Mol Cancer Res.

[37] Asano T, Tada M, Cheng S, Takemoto N, Kuramae T (2008). Prognostic values of matrix metalloproteinase family expression in human colorectal carcinoma.. J Surg Res.

[38] Houghton AM, Grisolano JL, Baumann ML, Kobayashi DK, Hautamaki RD (2006). Macrophage elastase (matrix metalloproteinase-12) suppresses growth of lung metastases.. Cancer Res.

[39] Hanai J, Mammoto T, Seth P, Mori K, Karumanchi SA (2005). Lipocalin 2 diminishes invasiveness and metastasis of Ras-transformed cells.. J Biol Chem.

[40] Sevenich L, Schurigt U, Sachse K, Gajda M, Werner F (2010). Synergistic antitumor effects of combined cathepsin B and cathepsin Z deficiencies on breast cancer progression and metastasis in mice.. Proc Natl Acad Sci USA.

[41] Sloane B, Yan S, Podgorski I, Linebaugh B (2005). Cathepsin B and tumor proteolysis: contribution of the tumor microenvironment.. Semin Cancer Biol.

[42] Watson CJ, Kreuzaler PA (2009). The role of cathepsins in involution and breast cancer.. J Mammary Gland Biol Neoplasia.

[43] Nozaki H (1987). Amino acid analysis of human von Willebrand factor fragments cleaved by porcine calpain II.. Tokai J Exp Clin Med.

[44] Moore JC, Murphy WG, Kelton JG (1990). Calpain proteolysis of von Willebrand factor enhances its binding to platelet membrane glycoprotein IIb/IIIa: an explanation for platelet aggregation in thrombotic thrombocytopenic purpura.. Br J Haematol.

[45] Terraube V, Marx I, Denis CV (2007). Role of von Willebrand factor in tumor metastasis.. Thromb Res.

[46] Nierodzik ML, Plotkin A, Kajumo F, Karpatkin S (1991). Thrombin stimulates tumor-platelet adhesion in vitro and metastasis in vivo.. J Clin Invest.

[47] Eisman R, Surrey S, Ramachandran B, Schwartz E, Poncz M (1990). Structural and functional comparison of the genes for human platelet factor 4 and PF4alt.. Blood.

[48] Boutte AM, Friedman DB, Bogyo M, Min Y, Yang L (2011). Identification of a myeloid-derived suppressor cell cystatin-like protein that inhibits metastasis.. Faseb J.

[49] Trotter MJ, Colwell R, Tron VA (2003). Thrombospondin-1 and cutaneous melanoma.. J Cutan Med Surg.

[50] Rodriguez-Manzaneque JC, Lane TF, Ortega MA, Hynes RO, Lawler J (2001). Thrombospondin-1 suppresses spontaneous tumor growth and inhibits activation of matrix metalloproteinase-9 and mobilization of vascular endothelial growth factor.. Proc Natl Acad Sci U S A.

[51] Dustin ML, Olszowy MW, Holdorf AD, Li J, Bromley S (1998). A novel adaptor protein orchestrates receptor patterning and cytoskeletal polarity in T-cell contacts.. Cell.

[52] Sinha P, Clements VK, Bunt SK, Albelda SM, Ostrand-Rosenberg S (2007). Cross-talk between myeloid-derived suppressor cells and macrophages subverts tumor immunity toward a type 2 response.. J Immunol.

[53] Connolly MK, Mallen-St Clair J, Bedrosian AS, Malhotra A, Vera V (2010). Distinct populations of metastases-enabling myeloid cells expand in the liver of mice harboring invasive and preinvasive intra-abdominal tumor.. J Leukoc Biol.

[54] Kim S, Takahashi H, Lin WW, Descargues P, Grivennikov S (2009). Carcinoma-produced factors activate myeloid cells through TLR2 to stimulate metastasis.. Nature.

[55] MacCoss MJ, McDonald WH, Saraf A, Sadygov R, Clark JM (2002). Shotgun identification of protein modifications from protein complexes and lens tissue.. Proc Natl Acad Sci U S A.

[56] Tabb DL, Fernando CG, Chambers MC (2007). MyriMatch: highly accurate tandem mass spectral peptide identification by multivariate hypergeometric analysis.. J Proteome Res.

[57] Ma Z-Q, Dasari S, Chambers MC, Litton MD, Sobecki SM (2009). IDPicker 2.0: Improved protein assembly with high discrimination peptide identification filtering.. J Proteome Res.

[58] Zhang B, Kirov S, Snoddy J (2005). WebGestalt: an integrated system for exploring gene sets in various biological contexts.. Nucleic Acids Res.

[59] Kirov SA, Zhang B, Snoddy JR (2007). Association analysis for large-scale gene set data.. Methods Mol Biol.

[60] Tazi KA, Quioc J-J, Abdel-Razek W, Tellier Z, Guichard C (2009). Protein array technology to investigate cytokine production by monocytes from patients with advanced alcoholic cirrhosis: An ex vivo pilot study.. Hepatol Res.

[61] Tabb DL (2008). What's driving false discovery rates?. J Proteome Res.

[62] Elias JE, Gygi SP (2007). Target-decoy search strategy for increased confidence in large-scale protein identifications by mass spectrometry.. Nat Methods.

[63] Vellaichamy A, Sreekumar A, Strahler JR, Rajendiran T, Yu J (2009). Proteomic interrogation of androgen action in prostate cancer cells reveals roles of aminoacyl tRNA synthetases.. PLoS ONE.

